# Motoric Mechanisms for the Emergence of Non-local Phonological Patterns

**DOI:** 10.3389/fpsyg.2019.02143

**Published:** 2019-09-26

**Authors:** Sam Tilsen

**Affiliations:** Department of Linguistics, Cornell University, Ithaca, NY, United States

**Keywords:** Articulatory Phonology, Selection-coordination theory, locality, phonology, harmony

## Abstract

Non-local phonological patterns can be difficult to analyze in the context of speech production models. Some patterns – e.g., vowel harmonies, nasal harmonies – can be readily analyzed to arise from temporal extension of articulatory gestures (i.e., spreading); such patterns can be viewed as articulatorily local. However, there are other patterns – e.g., nasal consonant harmony, laryngeal feature harmony – which cannot be analyzed as spreading; instead these patterns appear to enforce agreement between features of similar segments without affecting intervening segments. Indeed, there are numerous typological differences between spreading harmonies and agreement harmonies, and this suggests that there is a mechanistic difference in the ways that spreading and agreement harmonies arise. This paper argues that in order to properly understand spreading and agreement patterns, the gestural framework of Articulatory Phonology must be enriched with respect to how targets of the vocal tract are controlled in planning and production. Specifically, it is proposed that production models should distinguish between excitatory and inhibitory articulatory gestures, and that gestures which are below a selection threshold can influence the state of the vocal tract, despite not being active. These ideas are motivated by several empirical phenomena, which include anticipatory posturing before production of a word form, and dissimilatory interactions in distractor-target response paradigms. Based on these ideas, a model is developed which provides two distinct mechanisms for the emergence of non-local phonological patterns.

## Introduction

This paper addresses the topic of locality in the origins of phonological patterns. The main focus is on developing a model of speech production that is sufficient to generate non-local patterns. The conclusion is that even when non-local agreement relations between segments are observed, the mechanisms which gave rise to such relations can be understood to operate locally. This is desirable if we wish to avoid a conception of speech that allows for “spooky action at a distance,” i.e., discontinuities in the motor planning processes which determine the articulatory composition of word. It is important to note that the model developed here involves the planning and production of word forms by an individual speaker, and the articulatory patterns generated by the model are viewed as seeds of potential sound change on larger spatial and temporal scales. The starting point of the model is the gestural framework of Articulatory Phonology (Browman and Goldstein, 1989) and Task Dynamics (Saltzman and Munhall, 1989); recent extensions to this model in the Selection-coordination framework (Tilsen, 2016, 2018a,b) are also incorporated. We will develop an extension of these models in which there are two distinct ways for non-local patterns to arise; these mechanisms are shown to account for the origins of spreading and agreement harmonies, respectively.

In the gestural scores of Articulatory Phonology/Task Dynamics (henceforth AP/TD), an interval of gestural activation corresponds to a period of time in which there is force acting upon the state of the vocal tract, potentially driving it toward a new equilibrium value. Both the state parameter and the equilibrium value are typically represented by gestural labels in a score, e.g., an interval labeled as LA *clo* specifies the vocal tract state parameter as LA (lip aperture) and the equilibrium value as *clo*, i.e., a physical value corresponding to bilabial closure. Because of their inherent temporality, gestural activation intervals in the score provide a convenient proxy for mapping between a hypothesized cognitive system for control of movement and the empirical outputs of that system, i.e., changes in vocal tract states during speech. Yet there are many ways in which interpretation of the score necessitates familiarity with the underlying TD model. Indeed, there are several aspects of the system which are *not* shown in scores, and there are phenomena which scores are not well suited for describing.

**FIGURE 1:**
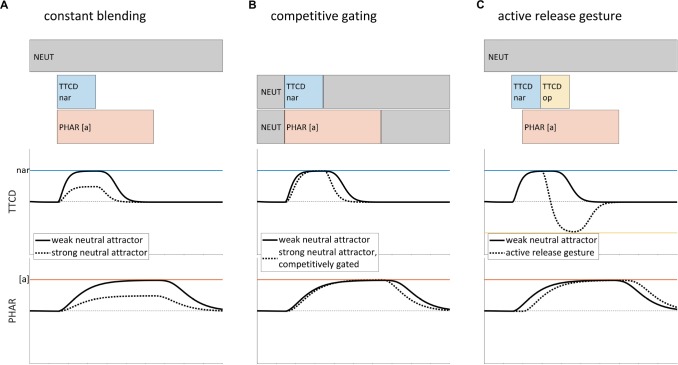
Sparseness of representation in the gestural score and the role of the neutral attractor, for a hypothesized production of [sa]. The solid lines in all examples show a weak neutral attractor resulting in a slow release of constriction. **(A)** Strong neutral attractor with constant blending results in target undershoot (dashed line). **(B)** Undershoot is avoided in the Task Dynamic model by competitively gating the influence of the neutral attractor. **(C)** Alternative model in which constriction release is accomplished by an active gesture.

To illustrate these points, we consider three issues in gestural representations of speech, which are relevant in different ways to our model of non-local patterns. The first issue is the role of the *neutral attractor*, which is hypothesized to govern the evolution of articulator states in the absence of gestural activation (Saltzman and Munhall, 1989; henceforth SM89). As we show below, there is a trade-off between the complexity of the neutral attractor and the postulation of additional gestures in the score. Figure 1 shows several versions of gestural scores for a CV syllable, [sa]. Below the scores are a couple of the relevant tract variables and gestural targets (here and elsewhere we omit some tract variables/gestures, such as glottal aperture, for clarity of exposition). The gestural activation intervals of the score are periods of time in which the driving force on a tract variable is influenced by a gesture. For example, the segment [s] corresponds to a [TTCD nar] gesture. When [TTCD nar] becomes active, the TTCD tract variable is driven toward the associated target (i.e., a value labeled as *nar*, which refers to a degree of constriction that is sufficiently narrow to generate audibly turbulent airflow). What is not conventionally specified in gestural scores is the mechanism that drives a release of that constriction. In the SM89 model, the neutral attractor drives model articulators toward default positions when there are no active gestures that influence those articulators; it has a direct influence on model articulator states, but only an indirect influence on tract variables. Importantly, the neutral attractor is not a “gesture” because it does not directly specify a target in tract variable coordinate space.

Of primary interest in the example is how to model interactions between influences of the neutral attractor and influences of gestural activation. For the sake of argument, let’s suppose – contrary to the SM89 model – that the effects of the neutral attractor on model articulator targets and stiffness (how quickly model articulators are driven to a target position) are blended with the effects of active gestures, and that the two neutral attractor blending strengths (i.e., stiffness blending and target blending) are correlated and constant throughout production of a word form. In this hypothetical situation, the model exhibits empirical deficiencies. Specifically, if the blending strength of the neutral attractor is relatively weak, then tract variables are slow to return to neutral positions after they have been displaced by gestural forces. For example, in Figure 1A, the hypothetical model exhibits an unrealistically slow release of the TTCD constriction (solid line). Simply strengthening the blended influence of the neutral attractor results in a different problem: the target of [TTCD nar] is never achieved (dashed line, Figure 1A). This target undershoot occurs because the relevant model articulators are driven to positions which reflect a compromise between the target of [TTCD nar] and the default positions associated with the neutral attractor. The empirical deficiencies associated with this hypothetical model are a consequence of the suppositions that stiffness and target blending strengths are related, and that the blending is constant.

The SM89 model does not presuppose that blending is constant. Instead, the SM89 model competitively gates the influence of the neutral attractor and the influences of gestures: when any active gesture influences a model articulator, the neutral attractor for that model articulator has no influence; conversely, when no active gestures influence a model articulator, the neutral attractor influences that articulator. This entails that the blending strength of the neutral attractor varies abruptly between minimal blending and maximal blending. The effect of competitive gating on tract variables is shown in Figure 1B. Competitive gating mitigates the problems that arise from constant blending: post-gesture releases are more rapid and target undershoot is avoided.

The neutral attractor gating mechanism (Figure 1B) appears to be empirically adequate, but to my knowledge there is no direct evidence that this is the correct conceptualization of the control system. Moreover, there is a subtle conceptual problem with the competitive gating mechanism: whereas the neutral attractor directly influences model articulators, active gestures only indirectly influence articulators, via their influences on tract variables. It may be somewhat worrying that a mechanism must be posited which is sensitive to gestural activation – i.e., forces on tract variables, but which affects the neutral attractor, which is not a force on tract variables. Another problem is that this mechanism may be overly powerful in its ability to abruptly shut off the neutral attractor for specific model articulators during production of word form.

A logical alternative to competitive gating is a model in which constriction releases are accomplished via active gestures, such as [TTCD op] (which releases the TTCD constriction). This has been proposed by several researchers and is sometimes called the split gesture hypothesis (Browman, 1994; Nam, 2007a, b; Tilsen and Goldstein, 2012). As shown in the score of Figure 1C, a [TTCD op] gesture can be active and appropriately phased relative to [TTCD nar], so as to drive a constriction release. Alternatively, [TTCD op] may be co-active with the vocalic [PHAR [a]] gesture, and gestural blending can modulate its influence during the period of time in which [TTCD nar] is active. In either case, the release of the TTCD constriction is sufficiently rapid (dashed line in TTCD panel). The point of contrasting the analyses in Figures 1B,C is to show that there is a trade-off between positing additional gestures and utilizing a more powerful blending mechanism. This is highly relevant to the model we develop below, which proposes a substantial expansion of the inventory of gestures and reconceptualizes the neutral attractor.

A closely related issue is that in many uses of gestural score representations, the velum and glottis are assumed to obtain default states during speech, in the absence of active velar or glottal gestures. The theoretical implications of this assumption have not been thoroughly examined in previous literature. The model we develop below does away with the notion of default states. Thus the reader should note that when velum or glottal gestures are omitted from scores in this paper, it is out of convenience/clarity, rather than a theoretical claim.

**FIGURE 2:**
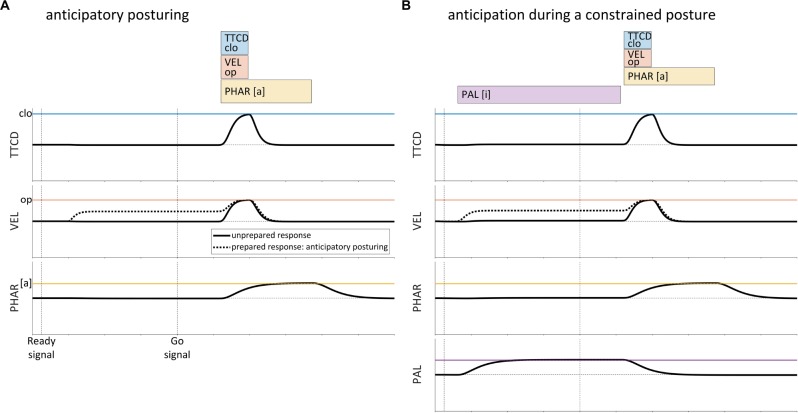
Schematic examples of anticipatory posturing effects in the production of the syllable/na/. **(A)** In a prepared response (dashed lined), the velum is partly open during the ready phase. A similar degree of opening is not observed in unprepared responses (solid line). **(B)** Anticipatory effects in prepared responses also occur when the posture of the vocal tract is constrained by the requirement to produce a prolonged vowel before the go-signal.

A second issue with gestural scores is that there are movements that occur prior to production of a word form which do not appear to be prototypically gestural. In particular, several studies have found evidence that speakers anticipatorily posture the vocal tract before producing an utterance, in a manner that is contingent upon the initial articulatory content of the utterance (Rastle and Davis, 2002; Kawamoto et al., 2008; Tilsen et al., 2016; Krause and Kawamoto, 2019a, b). For example, Tilsen et al. (2016) conducted a real-time MRI investigation in which CV syllables /pa/,/ma/,/ta/, and /na/ were produced in both prepared and unprepared response conditions. In the prepared response condition, the target syllable was cued together with a ready signal, which was followed by a variable delay (1250–1750 ms) prior to a go-signal. In the unprepared response condition, the target syllable was cued with the go-signal. Between-condition comparisons of vocal tract postures in a 150 ms period preceding the go-signal showed that in the prepared condition, many speakers adjusted the postures of their vocal organs in a manner that was specific to the upcoming response. This effect is schematized in Figure 2A, where the velum opens prior to the production of the syllable /na/.

Several aspects of anticipatory posturing effects are important to note here. First, the effects observed are predominantly assimilatory: anticipatory posturing almost always results in postures that are closer to the articulatory targets of the upcoming response. Second, effects are observed for a variety of tract variables/articulators, including lip aperture, tongue tip constriction degree, tongue body constriction degree, velum aperture, pharyngeal aperture, and vertical position of the jaw. Third, the effects are sporadic across speakers and articulators: not all speakers exhibit statistically reliable effects, and the tract variables in which effects are observed vary across speakers. Fourth, in an independently controlled condition in which speakers are required to maintain a prolonged production of the vowel [i] during the ready phase, anticipatory posturing effects are also observed. A schematic example of anticipatory posturing for /na/ while the posture of the vocal tract is constrained is shown in Figure 2B.

Notably, many of the anticipatory posturing effects observed in Tilsen et al. (2016) were *partial* assimilations: the ready phase posture in the prepared condition was only part of the way between the posture in the unprepared condition and the posture associated with achievement of the relevant gestural target. Furthermore, although not quantified in the study, it was observed that in prepared response conditions, the anticipatory movements that occurred in the ready phase exhibited slower velocities than movements conducted during the response.

Anticipatory posturing is challenging to account for in the standard AP/TD framework. The anticipatory movements cannot be attributed solely to a neutral attractor, because of their response-specificity: the neutral attractor would have to be modified in a response-contingent manner. The phenomenon also cannot be attributed solely to early activation of gestures: gestural activation should result in achievement of canonical targets, unless an *ad hoc* stipulation is made that pre-response gestures have alternative targets. A reasonable account is one in which the effects of anticipatorily activated gestures are blended with those of the neutral attractor; this would explain the partially assimilatory nature of the pre-response postures. However, recall from above that blending of the neutral attractor with active gestures is precisely what the SM89 model prohibits via the competitive gating mechanism (see Figure 1B), and this is necessary because an overly influential neutral attractor leads to the target undershoot problems illustrated in Figure 1A. Thus anticipatory posturing is something of a conundrum in the standard AP/TD framework.

A third issue with gestural scores is the representation of non-local agreement relations between gestures. Many theoretical approaches to phonology distinguish between “local” and “non-local” patterns (Pierrehumbert et al., 2000; Heinz, 2010; Rose and Walker, 2011; Wagner, 2012). Consider the hypothetical examples of harmonies in Table 1. Some languages exhibit co-occurrence restrictions in which certain consonants which differ in some particular feature do not occur in some morphological domain, such as a root or a derived stem. For example, (1) shows a sibilant anteriority harmony: all sibilants in a word form must agree in anteriority (i.e., alveolar vs. post-alveolar place of articulation). Consequently, [s] and [ʃ] cannot co-occur. Example (2) shows a pattern in which nasality spreads from a rightmost nasal stop to all preceding segments. Example (3) shows yet another pattern, nasal consonant harmony, in which coronal consonants must agree in nasality. The reader should consult the comprehensive survey of Hansson (2001) for a catalog of many real-language examples of consonant harmonies.

**TABLE 1:** Hypothetical examples of harmonies.

**(1) Sibilant harmony (spreading or agreement?)**	**(2) R -> L spreading of nasality (spreading)**	**(3) Nasal consonant harmony (agreement)**
a. sapas	a. nãmãn	a. sapas
b. ^∗^ʃapas	b. napas	b. ^∗^napas
c. ^∗^sapa ʃ	c. ^∗^nãpãn	c. ^∗^sapan
d. ʃ apa ʃ	d. ^∗^sãmãn	d. napan

There are two questions regarding these examples that are relevant here. First, how should articulatory patterns with non-local relations be represented in a gestural score, and second, what are the mechanisms which lead to their emergence on the timescale of utterances, for individual speakers? There is an ongoing debate regarding these questions. Gafos (1999) argued that many non-local patterns arise from gestural spreading, in which the activation of a gesture extends in time. Spreading of a feature or extended gestural activation is quite sensible for patterns such the one as example (2), where intervening segments show evidence of being altered by the spreading feature, nasality in this case. The spreading analysis may also be tenable when the effect of a temporally extended gesture does not result in drastic changes in the expected acoustic and/or auditory consequence of the intervening. For example, in the case of the sibilant harmony in example (1), a tongue tip constriction location gesture (i.e., [TTCL +ant] or [TTCL −ant]) may be active throughout the entirety of a word form without resulting in substantial acoustic effects: the TTCL gesture may have relatively subtle effects on intervening vocalic postures and is masked by non-coronal consonantal constrictions, such as an intervening bilabial closure. There is indeed some articulatory evidence for spreading that involves lingual postures (Walker et al., 2008; Whalen et al., 2011).

**FIGURE 3:**
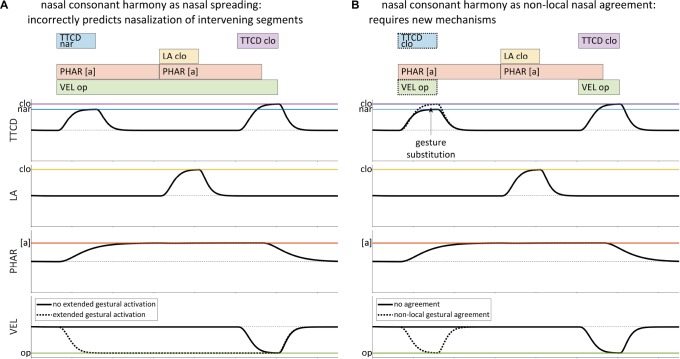
Comparison of gestural representations of spreading and non-local agreement for a harmonic pattern in which /sapan/ –¿ [napan]. **(A)** Extended activation of a velum closing gesture is empirically inadequate for representing nasal consonant harmony. **(B)** Nasal consonant harmony requires a mechanism which substitutes [VEL op] and [TTCD clo] gestures for [TTCD nar], when [VEL op] is present.

However, not all cases of harmony are readily amenable to a spreading analysis. A wide variety of consonant harmonies are reported in Hansson (2001), involving features such as voicing, aspiration, ejectivity, implosivity, pharyngealization, velarity, uvularity, rhoticity, laterality, stricture, and nasality. Hanson and others (Walker, 2000; Heinz, 2010) have argued that many of these patterns cannot be readily understood as feature spreading or extended gestural activation, because the expected acoustic consequences of spreading are not observed and may be physically incompatible with articulatory postures required by intervening segments. Consider hypothetical example of nasal consonant harmony shown in Table 1, example (3), variations of which are attested in many Bantu languages and in other, unrelated languages (see Hansson, 2001). An attempt to represent a pattern in which /sapan/ –¿ /napan/with extended activation of a [VEL op] gesture, as in Figure 3A, is problematic in several ways: it incorrectly predicts nasalized vowels, nasalization of the oral stop [p], and nasalized fricatives as opposed to nasalized stops. Hence the extended gestural activation in Figure 3A does not provide an empirically adequate analysis of nasal consonant harmony.

Instead of spreading, nasal consonant harmony would seem to require a mechanism which forces certain gestures to appear in certain places in the score, but only when other gestures are present. For example, it is possible to posit a representation such as in Figure 3B, where the relevant TTCD constriction gestures co-occur with a [VEL op] gesture, and where [TTCD nar] becomes [TTCD clo]. But the representation does not directly address a number of important questions, namely: what is the nature of the association between the TTCD gestures and the [VEL op] gesture, with respect to the knowledge of speakers? How do such co-occurrence restrictions arise on the scale of individual utterances? How can such patterns be productive in derived domains? The crux of the problem is that the AP/TD model offers no mechanism which can activate the [VEL op] gesture in precisely those circumstances which are consistent with the empirically observed harmony pattern.

This paper addresses the issues above and related ones by developing an extended model of articulatory control. The model incorporates two additional mechanisms of articulatory planning and substantially elaborates the standard model of Articulatory Phonology/Task Dynamics. Section “The Intentional Planning Mechanism” describes the first mechanism, *intentional planning*, where “intention” refers to a target state of the vocal tract. This mechanism involves the postulation of vocal tract parameter fields in which time-varying spatial distributions of activation are driven by excitatory and inhibitory input from gestures. The integration of activation in these fields determines a current target state of the vocal tract. Section “Gestural Selection and Intentional Planning” describes the second mechanism, *selectional planning*, in which gestures are organized into sets and the sets are organized in a hierarchy of relative excitation. Feedback-driven reorganizations of the excitation hierarchy generate an order in which sets of gestures are selected, executed, and suppressed. Crucially, selectional dissociations allow for individual gestures to be selected early or suppressed late, relative to other gestures. Neither of these mechanisms is novel: the intentional mechanism is borrowed from Dynamic Field Theory models of movement target representation (Schöner et al., 1997; Erlhagen and Schöner, 2002; Tilsen, 2007, 2009c; Roon and Gafos, 2016), and the selectional mechanism is borrowed from competitive queuing models of sequencing (Grossberg, 1987; Bullock and Rhodes, 2002; Bullock, 2004), which have been extended to model the selection of sets of gestures (Tilsen, 2016). However, the integration of these models in a gestural framework is somewhat new, having been first attempted in Tilsen (2009c) and more recently in Tilsen (2018b). The most novel contribution here is a reconceptualization of articulatory gestures that derives from integrating these frameworks. Specifically, we argue that it is useful to distinguish between two types of gestures: excitatory gestures and inhibitory gestures; furthermore, we claim that gestures which are non-active but nonetheless excited can influence the state of the vocal tract. Section “The Origins of Non-local Phonological Patterns” shows that with these hypotheses a new understanding of the origins of non-local phonological patterns is possible, one which is both motorically grounded and local. Crucially, our emphasis here is on the issue of origination/emergence/genesis: the mechanisms we develop create articulatory patterns in individual utterances for individual speakers, and these patterns are potential precursors of sound changes.

## The Intentional Planning Mechanism

An *intention* is, colloquially, an aim, purpose, goal, target, etc. Here we use *intentional planning* to refer to a mechanism which determines the target state of the vocal tract. It is important to note that this new conception of target planning requires us to maintain a distinction between *gestural targets* and the *dynamic targets* of the vocal motor control system. Instead of being fixed parameters of the speech motor control system, *dynamic targets* are states that evolve in real-time, under the influence of gestures, whose targets are long-term memories. The dynamic target states are modeled as integrations of activation in fields, drawing inspiration from previous models (Schöner et al., 1997; Erlhagen and Schöner, 2002; Tilsen, 2007). In this section we present a basic model of intentional planning and discuss evidence for the model.

### A Dynamic Field Model of Intentional Planning

**FIGURE 4:**
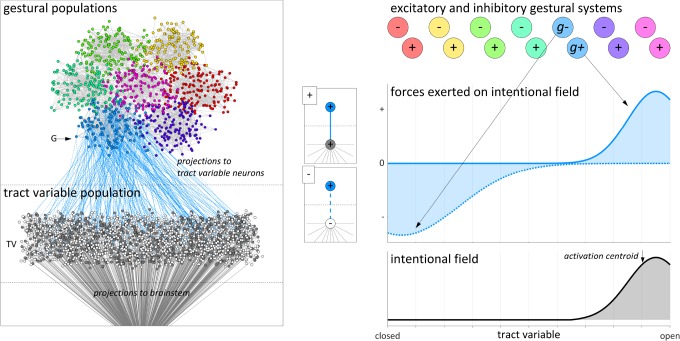
Microscale and macroscale visualizations of intentional planning model. Neurons in a gestural population (light blue) project to neurons in a tract variable population. The effects of these projections are conceptualized as excitatory and inhibitory forces exerted by gestures on an intentional planning field.

To develop intuitions for why a field model of intentional planning is sensible, we begin by elaborating a microscale conception of parameter fields, gestures, and their interactions. We imagine that there are two distinct types of populations of microscale units, tract variable (TV) populations and gestural (G) populations. For simplicity, Figure 4 depicts only a single TV population along with a small set of G populations. The microscale units are viewed as neurons, and we envision that there are both inhibitory and excitatory neurons in both types of populations. The inhibitory neurons only project locally, within populations. Each G population projects to one TV population, and multiple G populations may project to the same TV population. Each TV population is assumed to exhibit some degree of somatotopic organization, such that the neurons can be arranged in a one-dimensional space which maps approximately linearly to target values of some vocal tract parameter. The units in the TV population are assumed to project to brainstem nuclei which ultimately control muscle fiber tension. We assume that there is some degree of homotopic spatial organization in TV-to-brainstem projections, i.e., a projective efferent field analogous to receptive afferent fields of neurons in primary sensory cortices.

The post-synaptic targets of projections from G to TV populations provide a basis for distinguishing between excitatory and inhibitory forces in the macroscale conception of intentional planning. Consider that some of the neurons in a given G population project to excitatory neurons in the relevant TV population (depicted in Figure 4 as (+) projections), and others project to inhibitory neurons [i.e., (−) projections]. We conjecture that for a given G population there is a spatial complementarity between the distributions of these two types of projections. Thus a given G population preferentially excites the excitatory neurons in some region of the TV population and inhibits excitatory neurons in some other region (the inhibition occurs indirectly because the G population projects to inhibitory neurons, which in turn project locally to excitatory neurons within the TV population).

Given the above microscale conception, we construct a macroscale model in which the G populations are *gestural systems* (*g-systems*) and the TV populations are *intentional planning fields*. Furthermore, because of the distinction between (+) and (−) G-to-TV projections, we can conceptually dissociate a given gestural system into g+ and g− subsystems, i.e., subpopulations which excite and inhibit regions of an intentional field. Each g+ and g− system has a time-varying excitation value which is assumed to reflect a short-time integration of the spike-rate of the neurons in the population. The integrated effects of the projections from g-systems to the TV population are understood as forces acting on an intentional field. Microscopically the strengths of these forces are associated with the numbers of G-to-TV projections and their synaptic efficacies; on the macroscale the strengths of the forces are the product of g-system excitation and a weight parameter which represents the microscale connectivity and which is constant on the utterance timescale. The pattern of spatial activation in the intentional field is driven by these forces, and the activation centroid is hypothesized to determine a current target state for the vocal tract parameter. In other words, the dynamic target is an activation-weighted average of tract variable parameter values defined over an intentional planning field. Gestural system forces modulate the distribution of activation over intentional fields, but because the timescale of changes in G-to-TV synaptic connectivity and efficacy is relatively slow, gestural targets are best viewed as a long-term memory contribution to dynamic targets.

For concreteness, one can imagine that the relevant G population (light blue circles) in Figure 4 is associated with a [VEL op+] gesture, which exerts an excitatory force on the region of the velum aperture field that drives an opening of the velum. In addition, one can imagine that there is a [VEL op−] gesture which exerts an inhibitory force on the region of the field associated with closing the velum. There is a large amount of explanatory power that we obtain by dissociating the excitatory and inhibitory components of gestures in this way. Note that in the example of Figure 4, the inhibitory force is shown to have a broader distribution than the excitatory one, but more generally the relative widths and amplitudes of force distributions might vary according to many factors. Moreover, in the general case multiple g+ and g− systems may exert forces on the same intentional field, and this allows the model to generate a range of empirical phenomena. The reader should imagine that there are many of these fields, perhaps one for each tract variable of the task dynamic model, and that the fields are relatively independent of each other, at least to a first approximation.

For a generic implementation of intentional planning, the time-evolution of the state of each parameter field *u*(*x*,*t*) can be modeled numerically using a normalized coordinate *x* which ranges from 0 to 1 in small steps. Equation 1 shows three terms that govern the evolution of the field. The first is an activation decay term, with gain α, entailing that in the absence of input, *u*(*x*) relaxes to zero and that field activation saturates with strong excitatory input. The second term is the excitatory force, where *N* is a Gaussian function of *x* with mean μ*_*i*_*^+^ and standard deviation σ*_*i*_*^+^ associated with gesture *g*_*i*_. The term *G*_*i*_^+^ represents a gestural force gating function; it is modeled as a sigmoid function of the excitation value of gesture *g*_*i*_, and modulates the amplitude of the Gaussian force distribution. In typical cases, the sigmoid gating function is parameterized such that it only allows gestures with excitation values greater than some threshold value to exert substantial forces on an intentional field; however, we will subsequently explore the consequences of leaky gating, in which a gesture with an excitation value below the threshold can exert a substantial force on an intentional field. The gain term β^+^ controls the overall strength of the excitatory input. The third term is the inhibitory force, and its components mirror those of the excitation term. Note that excitatory and inhibitory force distributions may differ in their width (σ*_*i*_*^+^ vs. σ*_*i*_*^–^), and the condition *u*(*x*,*t*) ≥ 0 is imposed at each time step. Equation 2 shows the calculation of the dynamic target as the average activation-weighted parameter value, i.e., the field activation centroid.

$$\mathrm{Eq}\,.1\,\frac{\mathrm{du}\left(x\right)}{\mathrm{dt}}=\underset{\mathrm{decay}}{\underbrace{-\alpha \,u\,\left(x\right)}}+\underset{\mathrm{excitation}}{\underbrace{\beta^{+}\,\sum_{i}G_{i}^{+}\,𝒩\,\left(x,\mu_{i}^{+},\sigma_{i}^{+}\right)}}$$

$$+\underset{i\,n\,h\,i\,b\,i\,t\,i\,o\,n}{\underbrace{\beta^{-}\,\sum_{i}G_{i}^{-}\,𝒩\,\left(x,\mu_{i}^{-},\sigma_{i}^{-}\right)}}\;$$

$$\mathrm{Eq}\,.2T\left(t\right)=\frac{\sum_{x}x\;u\left(x,t\right)}{\sum_{x}\;u\left(x,t\right)}$$

The model equations above are used in all subsequent simulations and visualizations. These equations should be viewed as tools for describing phenomena on a relatively macroscopic scale, rather than constituting a definitive claim about a neural mechanism. Note that related but somewhat different equations have been presented in Tilsen (2007, 2018b).

### Empirical Evidence for Intentional Planning

The somatotopic organization of intentional planning fields provides a “spatial code” for movement target planning, i.e., a representation in which a spatial distribution in the nervous system encodes a target in the space of vocal tract geometry. One motivation for positing a spatial code of this sort comes from studies of manual reaching and eye movement trajectories using a distractor-target paradigm. In this paradigm, a participant is presented with a distractor stimulus and shortly thereafter a target stimulus; the participant then reaches or looks to the target. The distractor stimulus is understood to automatically induce planning of a reach/saccade to its location, and this planning is hypothesized to subsequently influence the planning and execution of the reach/saccade to the target location.

Both assimilatory and dissimilatory phenomena are observed in the distractor-target paradigm, depending on the proximity or similarity of the distractor and target. When the distractor and target stimulus are sufficiently proximal in space, or are associated with similar movements, there is an assimilatory interaction in planning: reaches and saccades to the target are observed to deviate toward the location of the distractor (Ghez et al., 1997; Van der Stigchel and Theeuwes, 2005; Van der Stigchel et al., 2006). In speech, the analogous phenomenon of distractor-target assimilation has been observed between vowels (Tilsen, 2009b): formants in productions of the vowel [a] were assimilated toward formants of a distractor stimulus which was a subcategorically shifted variant of [a]; likewise, assimilation was observed for [i] and a subcategorically shifted variant of [i].

**FIGURE 5:**
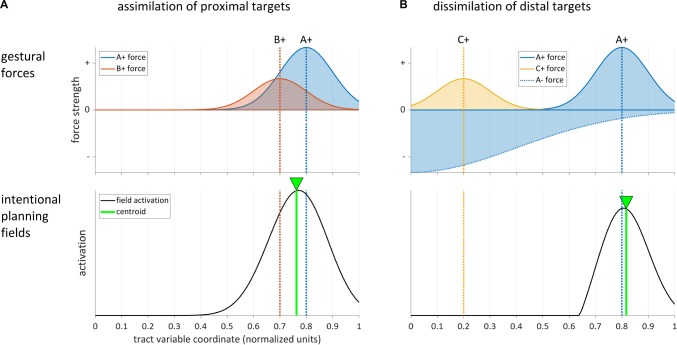
Dynamic field model simulations of assimilatory and dissimilatory effects in intentional planning. Top panels show gestural forces, bottom panels show field activation. **(A)** Assimilation of two gestures [A+] and [B+] with proximal targets. **(B)** Dissimilation between gestures with distal targets arises from a strong inhibitory force from gesture [A–].

Erlhagen and Schöner (2002) (cf. also Schöner et al., 1997) presented a dynamic field model capable of producing this assimilatory pattern (see also Tilsen, 2007, 2009a; Roon and Gafos, 2016). A simulation of the effect is shown in Figure 5A, where the target gesture is A+ and the distractor gesture is B+. Gesture-specific input to the field creates Gaussian distributions of excitatory forces on the parameter field. The dashed lines show the modes of the force distributions of A+ and B+. Because the targets of the gestures are similar or proximal in the field, they do not exert inhibitory forces upon one another. The activation of the intentional planning field represents a combination of these forces, and the centroid of activation (green line) is shifted from A to B in an assimilatory fashion.

In contrast to the assimilatory pattern, a dissimilatory pattern arises when the distractor and target are sufficiently distal in space or associated with different response categories. Eye movement trajectories and reaches are observed to deviate away from the location of the distractor in this case (Houghton and Tipper, 1994, 1996; Sheliga et al., 1994). In speech, the analogous effect was observed in Tilsen (2007, 2009b): vowel formants of productions of [a] were dissimilated from formants of [i] when an [i] distractor was planned, and vice versa. A similar dissimilation was observed in F0 measures between Mandarin tone categories in a distractor-target paradigm (Tilsen, 2013b). These dissimilatory phenomena have been explained by hypothesizing that inhibition of the region of the field activated by the distractor shifts the overall activation distribution so that its centroid is further away from the target than it would otherwise be in the absence of the inhibition (Houghton and Tipper, 1994). This can be modeled by assuming that the inhibitory force influences the region of the field which encodes the target. The effect is shown in Figure 5B, where [A+] is the target gesture, [C+] is the distractor, and [A−] is an inhibitory gesture which is coproduced with [A+]. The inhibitory force exerted by [A−] not only cancels the excitatory force of [C+], but also shifts the centroid of the activation distribution away from [C+], resulting in a subtle dissimilation. Note that in order for this effect to arise, the inhibitory force distribution has to be either wide enough to overlap with the excitatory one, or its center has to be sufficiently close to the center of the excitatory one. Tilsen (2013b) argued that dissimilatory effects of this sort may be pervasive and provide a motoric mechanism for the preservation of contrast. In this view, degrees of resistance to coarticulation (Recasens, 1985; Fowler and Brancazio, 2000; Cho et al., 2017) might be understood as manifested by gradient differences in the amplitudes and widths of inhibitory gestural forces.

Another form of evidence for intentional planning is anticipatory posturing effects of the sort described in section “Introduction,” Figure 2. There we noted that speakers exhibit vocal tract postures that are partially assimilated to the targets of gestures in an upcoming response. This phenomenon shows that some gesture-specific influences on the state of the vocal tract are present, even before a gesture becomes “active” (in the standard AP/TD sense). Discussion of how such effects are modeled in the current framework is deferred to section “Sub-selection Intentional Planning and Anticipatory Posturing,” after we have presented a mechanism for organizing the selection of gestures.

### The Inadequacy of Gestural Blending

The Articulatory Phonology/Task Dynamics (AP/TD) model cannot readily generate assimilatory or dissimilatory effects of the sort described above. A key point here is that in the distractor-target paradigm, only one of the tasks – the one associated with the target stimulus – is actually executed. This entails that only the target gesture becomes active, not the distractor. Of course, if both gestures were active, their influences on the target state of the vocal tract could be blended, resulting in an intermediate target. This blending is accomplished by a making the current target of a tract variable a weighted average of active gestural targets (Saltzman and Munhall, 1989). For example, if [A] and [B] have targets of T_*A*_ = 0 and T_*B*_ = 1 and blending weights of w_*A*_ = w_*B*_ = 0.5, the blended target T = (T_*A*_w_*A*_ + T_*B*_w_*B*_)/(w_*A*_ + w_*B*_) = 0.5, which is an intermediate value between T_*A*_ and T_*B*_. The problem is that if only the target gesture is produced, the distractor gesture never becomes active, and the weight of [B] should be 0. Hence it is necessary to incorporate a mechanism whereby gestures which are not active can influence the dynamic targets of the vocal tract. We pursue this in section “Gestural Selection and Intentional Planning.”

With regard to dissimilatory effects, the standard view of gestural blending is even more problematic. In order for blending of simultaneously active gestures to generate dissimilation, the calculation of a tract variable target must allow for negative weights. For example, if [A] and [B] have targets T_*A*_ = 0 and T_*B*_ = 1, and blending weights w_*A*_ = 0.5 and w_*B*_ = −0.1, then T = 1.25. This seems somewhat problematic from a conceptual standpoint because the blending function is undefined when w_*A*_ = −w_*B*_, and because it generates a hyper-assimilatory target when −w_*B*_ > w_*A*_. The problem of non-contemporaneous activation mentioned above also applies: the gesture of the distractor stimulus is not actually active; thus its weight should be 0 and it should not contribute to the calculation of the target.

As shown in section “Empirical Evidence for Intentional Planning,” a model of target planning in which the inhibitory and excitatory effects of gestures are dissociated and have spatial distributions over a field can readily accommodate both assimilatory and inhibitory patterns. This reinforces the idea that rather than thinking of a gesture as having a monolithic influence on the target state of the vocal tract, we can more usefully think of gestures as having two distinct components: an excitatory component which exerts an excitatory force on a planning field, and an inhibitory component which exerts an inhibitory force on the same planning field. The temporal dynamics of activation of these two components of “the gesture” may in typical circumstances be highly correlated, but not necessarily so. It is logically possible and useful in practice to dissociate the exhibitory and inhibitory components. Thus the Articulatory Phonology conception of “a gesture” is re-envisioned here as a pair of gestures, one exerting an excitatory influence on a tract variable parameter field, the other exerting an inhibitory influence on the same field. For current purposes, we assume that the spatial distributions of the excitatory and inhibitory forces are effectively complementary, in that there is a single mode of the inhibitory distribution and this mode is distant from the mode of the excitatory distribution. More general force distributions may be possible, but are not considered here.

It important to clarify that the intentional planning model does not supplant the Task Dynamic model equations for tract variables and model articulators. In the TD model each tract variable *x* is governed by a second order differential equation: $\frac{1}{k}\,\ddot{x}+\frac{\beta }{k}\,\dot{x}+x=T\,\left(t\right)$, where *T*(*t*) is a dynamic target calculated by blending gestural targets. The equation is analogous to a damped mass-spring system, where the dynamic target *T*(*t*) is a driving force, and changes in *T* can be conceptualized as changes in the equilibrium length of the spring. The intentional planning mechanism proposed here merely supplants the Saltzman and Munhall (1989) blending mechanism and introduces a new type of gesture – an inhibitory gesture – which can influence the dynamic target.

However, in order to account for how gestures which are not contemporaneously active can have effects on the target state of the vocal tract, further revision of the AP/TD model is necessary. This requires an explicit model of when gestures may or may not influence intentional fields, and is addressed in the following sections.

## Gestural Selection and Intentional Planning

The gestural scores of Articulatory Phonology/Task Dynamics do not impose any form of grouping on the gestures in a score. Indeed, there is no direct representation of syllables or moras in standard gestural scores, and this raises a number of challenges for understanding various typological and developmental phonological patterns (see Tilsen, 2016, 2018a). In order to address these challenges, the Selection-coordination model was developed in a series of publications (Tilsen, 2013a, 2014a,b, 2016, 2018b). The Selection-coordination model integrates a competitive queuing/selection mechanism (Grossberg, 1987; Bullock and Rhodes, 2002; Bullock, 2004) with the coordinative control of timing employed in the AP/TD model. Because the selection-coordination model has been presented in detail elsewhere, only a brief introduction to the model is provided below. Furthermore, discussion of the full range of phonological patterns which the model addresses is beyond the scope of the current paper, and the reader is referred to other work for more thorough exposition (Tilsen, 2016, 2018a,b). Here we present the model in sufficient detail for the reader to understand how it interacts with intentional planning, and we address the question of when gestures may or may not influence intentional fields.

### The Organization of Gestural Excitation

**FIGURE 6:**
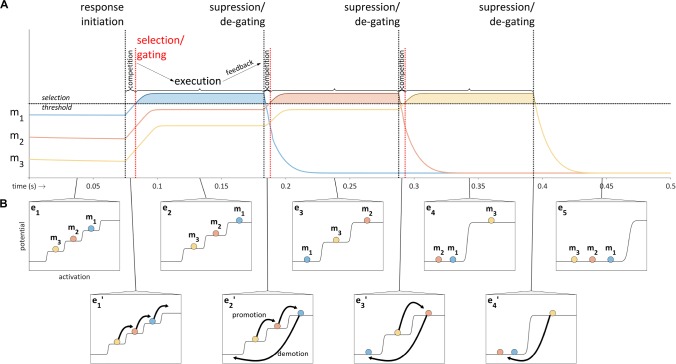
Schematic illustration of the competitive queuing model. **(A)** Sequencing of motor plans (m_1_, m_2_, and m_3_) is accomplished through a cycle of competition, execution, and feedback-induced suppression. Activation time-series are shown for each plan; vertical lines indicate response initiation and feedback-related selection/suppression events. **(B)** Excitation potential model of competitive queuing dynamics in which epochs of steady state relative excitation (e_1_–e_5_) are interrupted by abrupt reorganizations (e_1_’–e_4_’).

The selection-coordination model employs a mechanism for competitively selecting sets of gestures. The mechanism is based on a model of action sequencing developed in Grossberg (1987) which is referred to as *competitive queuing* (Bullock and Rhodes, 2002; Bullock, 2004). A key aspect of the competitive queuing model is that the plans for a sequence of actions are excited in parallel prior to and during production of the sequence, an idea which was advocated by Lashley (1951) and for which a substantial body of evidence exists (e.g., Sternberg et al., 1978, 1988). A schematic illustration of competitive queuing of three sets of motor plans – m_1_, m_2_, and m_3_ – is provided in Figure 6. Prior to response initiation, the plans have a stable relative excitation pattern; upon response initiation a competition process occurs in which the excitation of the plans increases until one exceeds a selection threshold. The selected plan (here m_1_) is executed while its competitors are temporarily gated. Feedback regarding achievement of the targets of the selected plan eventually induces suppression of that plan and degating of the competitors, at which point the competition process resumes, leading to the selection of m_2_. The cycle of competition, execution, and suppression iterates until all plans have been selected and suppressed.

The Selection-coordination theory hypothesizes that the motor plans of the competitive queuing model in Figure 6A can be viewed as sets of gestures in the context of speech production. When a given set of gestures is above the selection threshold, the gestures in that set are *selected*. Within each selected set, the timing of gestural activation/execution is controlled by phasing mechanisms which we do not address here. Hence selection of a gesture does not entail immediate activation of that gesture: coordinative phasing mechanisms of the sort hypothesized in the coupled oscillators model are assumed to determine precisely when selected gestures become active (Tilsen, 2016, 2018b). In many cases, and in particular for adult speakers in typical contexts, it makes sense to associate the aforementioned motor plan sets with syllables. Thus the selection-coordination model partitions multisyllabic gestural scores into a sequence of competitively selected scores.

In order to facilitate conceptualization of the competitive selection mechanism, the relative excitation pattern of the gestures in a set can be viewed as organized in a step potential, which has the effect of transiently stabilizing excitation values between periods of competition/suppression. This leads to the picture in Figure 6B, where abrupt reorganizations (e_1_′–e_4_′) intervene between stable epochs of organization (e_1_–e_5_). These reorganizations are understood to consist of promotion and demotion operations on gestures. Promotion increases excitation to the next highest level, and demotion lowers excitation of selected gestures to the lowest level. The topmost level of the potential is called the *selection level*, and the set of gestures which occupy the selection level are *selected*. Note that in order to avoid terminological confusion, we use the term *excitation* to refer a quantitative index of the states of gestural systems; the term *activation* is reserved to describe a state in which a gestural system exerts its maximal influence on an intentional planning field – this terminological distinction maintains some consistency with the Articulatory Phonology interpretation of gestural activation intervals in a gestural score. Importantly, gestures which are neither active nor selected can have gradient degrees of excitation which are below the selection threshold.

**FIGURE 7:**
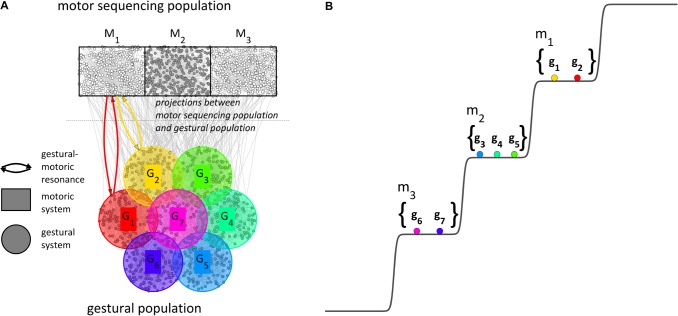
Microscale and macroscale conceptualizations of the motor sequencing population and gestural population. **(A)** The motor sequencing population differentiates into subpopulations which are conceptualized macroscopically as motoric systems; lexical memory determines a pattern of resonance between motoric systems and gestural systems. **(B)** The pattern of relative excitation of gestural systems is governed by a step potential, according to their associations with motoric systems.

We motivate the macroscopic model of Figure 6B from the microscopic picture in Figure 7A. In addition to populations of microscale units for gestural systems and tract variable parameters (not shown), we imagine a motor sequencing population. The motor sequencing and gestural populations have projections to one another, and the relevant projections are from excitatory neurons to excitatory neurons. When a word form is excited by conceptual/syntactic systems^1^ (or “retrieved from lexical memory”), the gestures associated with the word form become excited. The mutually excitatory projections between gestural and motoric populations give rise to resonant states which augment gestural system excitation. Crucially, it is conjectured that the motoric population differentiates into subpopulations which correspond to sets of gestures, i.e., motor systems (henceforth m-systems). It is assumed that the long-term memory of a word form^2^ includes information which determines the pattern of m-system differentiation, the pattern of resonances between g- and m-systems, and coupling relations between m-systems which are selected together. In the current example, the word form is comprised of three CV syllables and hence the motor population differentiates into three uncoupled, competitively selected m-systems (Figure 7B). If the excited word form were comprised of a different number of CV m-systems, the motor sequencing population would differentiate into that number. For syllables with a coda, diphthong, or long vowel, two anti-phase coupled m-systems would be organized in the same level of the potential.

The reader should note that the motor population differentiation pattern in Figure 7A exhibits a particular spatial arrangement, such that the initial m-system organization for a word form corresponds to the spatial pattern of differentiation in the motoric population. This spatial correspondence is not necessary for our current aim – modeling long-distance phonological patterns – but it is useful for a more comprehensive model in which the directionality of metrical-accentual patterns can be interpreted (see Tilsen, 2018a). Furthermore, it is important to emphasize that the motor population is finite and thus when a word form requires a greater number of m-system differentiations, the size of each m-system population becomes smaller and m-systems become more susceptible to interference. Thus an upper-bound on the number of simultaneously organized m-systems falls out naturally from the model, based on the idea that interference between m-systems destabilizes the organization (see Tilsen, 2018b).

One important advantage of the conceptual model is that the gestural-motoric resonance mechanism (g–m resonance) offers a way for gestures to be *flexibly* organized into syllable-sized or mora-sized units. Rather than resulting from direct interactions between gestures, syllabic organization arises indirectly from a pattern of resonances between g-systems and m-systems, in combination with the organization of m-systems into levels of relative excitation. In other words, g-systems interact not with each other, but instead couple with m-systems. These m-systems then couple strongly in stereotyped ways, giving rise to various syllable structures. This indirect approach to organization is desirable because direct interactions between g-systems are in conflict between word forms which organize the same gestures in different orders (e.g., *pasta* vs. *tapas*). Another advantage of the flexible organization based on g-m resonance is that it allows for developmental changes in the composition of m-systems, evidence of which is discussed in Tilsen (2016).

A final point to emphasize about the selection model is that the conception described above should be understood as a *canonical* model of a system state trajectory for sequencing, where “canonical” implies a standard against which other trajectories can be usefully compared. In the canonical trajectory, the relative excitation of sets of gestures is iteratively reorganized solely in response to external sensory feedback, and the reorganizations generate an order of selection which matches the initial relative excitation hierarchy. This trajectory serves as a reference for more general system state trajectories, for example ones in which reorganizations are not necessarily driven by external sensory feedback. Indeed, there is a particular form of deviation from the canonical trajectory which is highly relevant for current purposes. This deviation involves the use of internal rather than external feedback to govern reorganization; as we consider below, internal feedback allows for operations on the gestures in a set to be dissociated from each other.

### Selectional Dissociation and Local Coarticulation

An important aspect of the Selection-coordination model is that internal feedback can be used to anticipatorily select a gesture, before all of the gestures in the preceding epoch are suppressed. A great deal of evidence indicates that in addition to external sensory feedback, the nervous system employs a predictive, anticipatory form of feedback, called *internal feedback* (Wolpert et al., 1995; Kawato and Wolpert, 1998; Kawato, 1999; Desmurget and Grafton, 2000; Hickok et al., 2011; Parrell et al., 2018, 2019a,b). In the Selection-coordination model, if degating (i.e., promotion) and suppression (i.e., demotion) are contingent solely on external feedback, then there is necessarily a gap in time between target achievement of a preceding gesture and selection of a competitor gesture. However, if internal feedback is used to degate the competitor prior to target achievement of the preceding gesture, the gestural selection intervals can overlap. Pervasive overlap observed in spontaneous conversational speech indicates that anticipation/prediction of target achievement may be generally more influential on degating and suppression than the peripheral sensation of achievement, at least in adult speech. It might also be expected that the internal regime of control would be associated with less variability in the timing of selection than the external one, because external sensory information may be perturbed by contextual effects on movement targets or other environmental influences.

**FIGURE 8:**
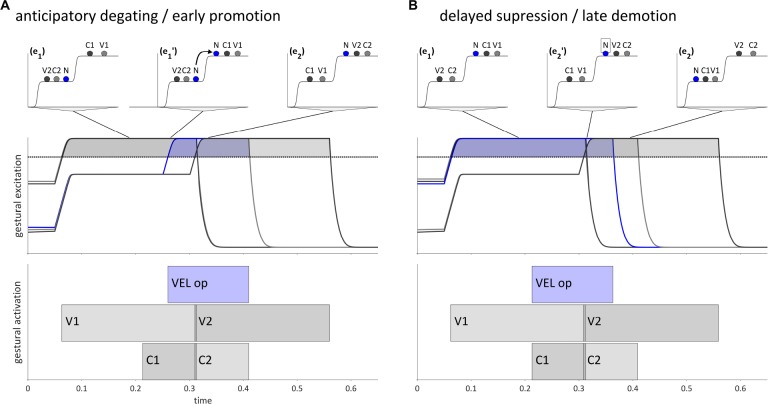
Dissociation of gestural promotion and demotion for intervocalic consonant-nasal sequences, VCNV and VNCV. **(A)** Anticipatory degating of a nasal gesture in a {VC}{NV} word form. **(B)** Delayed suppression of a nasal gesture in a {VN}{CV} word form. Lines from potentials indicate when in time a given pattern of activation occurs. Horizontal dashed lines are the selection threshold.

Internal feedback allows for dissociations of degating and suppression of gestures which are canonically selected in a given epoch. These *selectional dissociation* phenomena are illustrated in Figures 8A,B, which depict hypothesized trajectories for {VC}{NV} and {VN}{CV} word forms, respectively (V = vocalic gesture, N = velum opening gesture; C = oral constriction gesture). Specific phonological forms which instantiate these would be /eb.na/ and /en.ba/. The pattern in Figure 8A is an example of anticipatory degating, which we will also refer to as *early promotion*. The velum opening gesture ([VEL op], labeled “N” in the potentials), is associated with the second syllable, i.e., the second of two competitively selected m-systems. The oral constriction gesture associated with N is C2. In a canonical trajectory, there would be two distinct selection epochs, (e_1_) and (e_2_), and N would be promoted along with C2 in (e_2_), subsequent to suppression of V1 and C1. However, internal feedback anticipates target achievement of V1 and C2, and thereby allows N to be degated early and promoted. This results in there being a period of time (e_1_′) in which the [VEL op] gesture is selected along with gestures of the first syllable, resulting in a phonetic realization in which the stop is partially nasalized, i.e., [ebna] or [ebmna].

Conversely, Figure 8B shows a trajectory for a {VN}{CV} word form in which the [VEL op] gesture is suppressed late relative to gestures in the first syllable. In a canonical trajectory, [VEL op] would be demoted in the reorganization from (e_1_) to (e_2_). By hypothesis, reliance on internal feedback can not only anticipate target achievement, but also fail to anticipate target achievement, thereby creating a delay in the suppression of N relative to other gestures in the syllable, including the oral constriction gesture it is associated with, C1. This results in a period of time during which both [VEL op] and gestures associated with the second syllable are selected in (e_2_′), which gives rise to a phonetic form with a partially nasalized stop, i.e., [enb̃a] or [enmba].

The mechanisms of early promotion (anticipatory degating) and late demotion (delayed suppression) generate local assimilatory patterns. The early promotion in Figure 8A can be phonologized as the assimilation /VC.NV/→/VN.NV/(/ebna/→/emna/), and the late demotion in Figure 8B as /VN.CV/→/VN.NV/ (/enba/→/enma/). Here “phonologization” entails that selection of [VEL op] in both epochs of the word form occurs because long term (i.e., lexical) memory specifies that this is the case.

The selectional dissociation mechanism is potentially quite powerful, especially if it is unconstrained. An important question is: what prevents early promotion and late demotion from occurring pervasively and for extended periods of time? A generic answer to this question is that anticipatory degating and delayed suppression may be opposed by other mechanisms when they substantially alter the external sensory feedback associated with a word form and have adverse consequences for perceptual recoverability (see Chitoran et al., 2002; Chitoran and Goldstein, 2006; Tilsen, 2016). In particular, the degree to which the sensory alteration affects the perceptual distinctiveness of gestures should correlate with resistance to selectional dissociations. Ultimately, whether anticipatory degating and delayed suppression will be extensive enough to be phonologized as anticipatory or perseveratory assimilation must depend on a complex interplay of factors that includes the perceptual contrasts in a language along with occurrence frequencies of sets of gestures and their functional loads.

**FIGURE 9:**
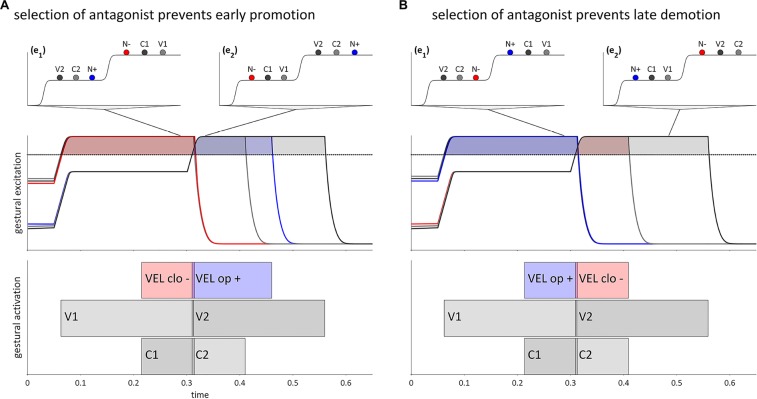
Selection of antagonistic gestures prevents selectional dissociation of the velum closing gesture in VCNV and VNCV forms. **(A)** Selection of [VEL clo–] in (e_1_) prevents early promotion of [VEL op+]. **(B)** Selection of [VEL clo–] in (e_2_) prevents late demotion of [VEL op+]. Lines from potentials indicate when in time the potential occurs.

A more specific source of restriction on selectional dissociation is hypothesized as follows. Given an excitatory gesture [x+], dissociated selection of [x+] is prevented if a gesture [y−], which is antagonistic to [x+], is selected. For example, [VEL clo−] is antagonistic to [VEL op+] because [VEL clo−] exerts a strong inhibitory force on the region of the velum aperture intentional field that [VEL op+] most strongly excites. The supposition here is that the selection of a gesture which is antagonistic to another gesture prevents the anticipatory degating or delayed suppression of that gesture. Figures 8, 9A,B show hypothetical examples of VCNV and VNCV, respectively. These could be instantiated specifically as forms /ebna/ and /enba/. In Figure 9A, selection of a [VEL clo−] gesture (shown as N− in the potential) in epoch (e_1_) opposes extensive anticipatory degating of [VEL op+] (N+ in the potential), and thereby prevents early promotion. Along the same lines, in Figure 9B selection of [VEL clo−] in (e_2_) prevents delayed suppression of [VEL op+] and thereby prohibits late demotion.

It is possible to hypothesize an even stronger restriction, in which an antagonistic pair of gestures can never be co-selected. In that case, an NV syllable such as [na] would correspond to a set of gestures in which [VEL op+] and [VEL clo+] are selected, but not [VEL op−] and not [VEL clo−]. In that case, blending of the co-selected [VEL clo+] and [VEL op+] gestures can generate an empirically adequate pattern of velum aperture for a nasal consonant-oral vowel syllable. Interestingly, any /NV/ syllable in this account would be necessarily be “underspecified” for inhibitory VEL gestures, which would make it more prone to being influenced by gestural dissociations. For current purposes, this stronger hypothesis prohibiting co-selection of antagonistic gestures is unnecessary: we only need the weaker hypothesis that selection of an inhibitory antagonist in some epoch prevents a selectional dissociation in which an excitatory gesture is selected in that same epoch.

### Sub-Selection Intentional Planning and Anticipatory Posturing

Here we integrate the intentional planning mechanism with the model of gestural selection described above. The basic question to address is: when is gestural excitation expected to result in observable changes in the state of the vocal tract? Given the model of intentional planning presented in section “The Intentional Planning Mechanism,” we can rephrase this as the question of when gestures exert forces on intentional planning fields. One answer which can be rejected is that intentional planning is only influenced by *active* gestures, i.e., gestures which have been selected and triggered by phasing mechanisms. Such an account would be natural in the standard AP/TD framework, but falls short empirically because it cannot straightforwardly generate anticipatory posturing effects or assimilatory/dissimilatory effects in distractor-target paradigms. Merely allowing gestural activation to vary continuously does not solve this problem because the standard model requires some mechanism to trigger a change from zero to non-zero activation.

Recall from section “Introduction” that a number of studies have provided evidence that speakers exert control over vocal tract posture prior to production of a word form, and do so in a way that is specific to gestures in the word form (see Figure 2). Analyses of discrepancies between acoustic and articulatory measurements of verbal reaction time in delayed response paradigms have provided indirect evidence for changes in vocal tract state prior to the initiation of movement (Rastle and Davis, 2002; Kawamoto et al., 2008). Direct evidence of response-specific anticipatory posturing was observed in the real-time MRI study designed specifically to test for such effects (Tilsen et al., 2016), discussed in section “Introduction.” This study showed that prior to the cued initiation of a response, speakers often adopted a vocal tract posture that was partly assimilated to upcoming gestural targets. Another recent study has shown that in a delayed word-naming task, speakers configure their lips to anticipate the initial consonantal articulatory target of a response, even when the complete gestural composition of the response is unknown (Krause and Kawamoto, 2019a).

A standard gestural activation account could, in principle, generate anticipatory posturing effects, but only with several *ad hoc* adjustments. First, the relevant gesture(s) would need to be allowed to become active prior to other gestures. Second, and more problematically, the anticipated gestures would need to have alternative targets, because the observed anticipatory posturing effects are partial. But in the standard AP/TD model each gesture is associated with a single target parameter; thus it is not entirely sensible to say that a single gesture is associated with two targets, one for anticipatory posturing and the other for normal production. Alternatively, the competitive gating of neutral attractor and gestural influences on model articulators (see Figure 1B) could be relaxed to allow for partial blending of these influences before production. Yet this would require a fairly *ad hoc* stipulation that only some model articulators are subject to the blending; moreover, the blending would need to be turned off (i.e., competitively gated) during production of the word form, otherwise target undershoot would be pervasive, as discussed in section “Introduction.”

**FIGURE 10:**
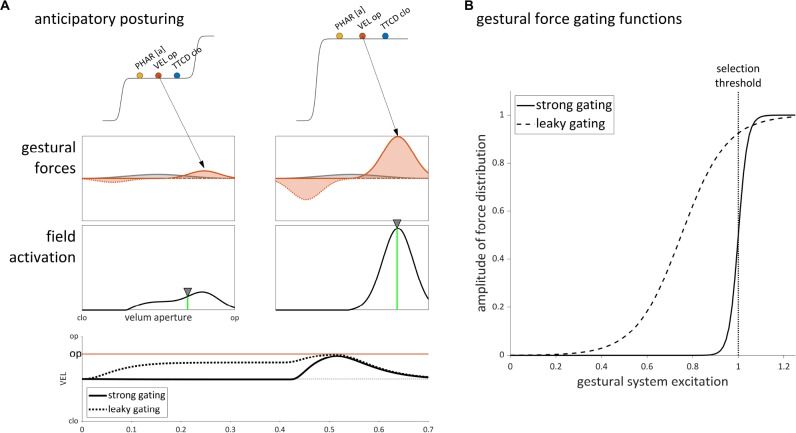
Sub-selection threshold influence of gestural excitation on the velum aperture tract variable field in production of the syllable /na/. **(A)** Anticipatory posturing arises from leaky gating of a [VEL op+] gesture. The effect on the VEL tract variable is shown in the bottom panel. **(B)** Comparison of gestural force gating functions with strong gating and leaky gating.

The selection-coordination-intention framework provides an alternative account of anticipatory posturing, based on the idea that gestural systems with excitation values below the selection threshold do in fact exert forces on intentional planning fields. Figure 10A illustrates this effect for velum opening in the syllable /na/, which is comprised of [TTCD clo±], [PHAR [a]±], and [VEL op±] gestures. Prior to overt production, the gestural systems are excited but below the selection threshold. Despite not being selected, the [VEL op±] gestures exert excitatory and inhibitory forces on the velum aperture intentional planning field. The excitatory force corresponds to a Gaussian distribution of activation in the field, indicated by the arrow. Note that a constant neutral attractor force on the field is also assumed to be present.

The amplitude of the gestural force distribution is modeled as a sigmoid function of the excitation value of [VEL op+] (see section “A Dynamic Field Model of Intentional Planning,” Eq. 1). Two differently parameterized sigmoid functions are shown in Figure 10B. The strong gating function changes abruptly from 0 to 1 in the vicinity of the selection threshold, resulting in negligible forces from gestures below the threshold, and in maximal forces from gestures which are selected. The leaky gating function is parameterized so that its midpoint is lower and its slope is shallower; this results in a non-negligible force being exerted on the velum aperture field, even when [VEL op+] has below-selection-level excitation. Either parameter of the sigmoid function (i.e., its midpoint or slope) can be adjusted to achieve this effect.

The difference between the strong and leaky gating functions is reflected in the tract variable time series shown in Figure 10A. With strong gating (solid line), the neutral attractor is the only substantial influence on the velum aperture field prior to gestural selection, and hence the tract variable remains in a neutral position. With leaky gating (dashed line), the [VEL op+] gesture exerts a substantial influence that drives the tract variable to an intermediate state. This pre-response anticipatory posturing effect results in only a partial assimilation because the dynamic target of the system (the weighted average of field activation) integrates both the neutral attractor influence and the influence of [VEL op+].

It is worth noting that leaky gating can generate both anticipatory and perseveratory posturing effects: subsequent to a production, a gesture with leaky gating can have a persistent influence on the state of the vocal tract, as long as the excitation of the gesture is not too low. The empirical characteristics of anticipatory posturing effects can thus be modeled fairly straightforwardly, as long as the parameters of the gating function are allowed to vary from gesture to gesture, speaker to speaker, and even from utterance to utterance. Of course, there may be a number of factors that can predict variation in the magnitude of such effects, and these are worth future investigation.

The above model suggests that a disambiguation of the phrase *gestural initiation* is in order. Gestures are “initiated” in two senses: gestures conceptualized as systems become excited, to some subthreshold degree, and this “initiation of excitation” may or may not result in observable effects on the state of the vocal tract, depending on the parameterization of the gating function. Subsequently, gestural systems are selected, i.e., their excitation exceeds a threshold, and when triggered by phasing mechanisms they can begin to exert their maximal influence on an intentional field, which constitutes an “initiation of activation.” At the same time, it is important to keep in mind that active gestures which influence the same tract variable can be blended, as in the standard AP/TD model, and thus activation of a gesture does not necessarily entail an immediately observable effect on the vocal tract.

In the context of the selection-coordination-intention framework, there is a potential ambiguity with regard to whether a given phonological pattern arises from selectional dissociations (i.e., early promotion/late demotion) or from subthreshold gestural forces allowed by leaky gating. Anticipatory and perseveratory phenomena might logically be understood to result from internal feedback-driven changes in gestural selection, or from changes in the parameterization of gating functions, or from a combination of both mechanisms. The question of which of these analyses to apply in a given context is explored in the next section, where we apply the model to understand non-local phonological patterns.

## The Origins of Non-Local Phonological Patterns

The selection and intention mechanisms provide two ways for the articulatory precursors of non-local phonological patterns to arise in individual utterances. It is important to emphasize that our primary aim here is a model of how non-local patterns (i.e., harmonies) *originate*. The issue of how such patterns are phonologized, i.e., become part of a phonological grammar, is a more general one, and treatment of this topic is beyond the scope of this paper. For current purposes, we assume an Ohalan conception of phonologization in which motoric mechanisms are bias factors that perturb articulatory realization, and in which these perturbations can be phonologized through hypocorrective mechanisms (Ohala, 1993). Hence the mechanisms presented below should be understood as operating on the timescale of a single utterance and the spatial scale of an individual speaker, but their effects may lead to change in behavior on larger temporal and spatial scales. Specifically, one can imagine that in a population of speakers there is stochastic variation in the parameters associated with various control mechanisms of the model (e.g., the leakiness of gating). Interactions between speakers may on supra-utterance time scales lead to population scale changes in such parameters, although this must be seen as a highly chaotic process which cannot be readily predicted. In any case, it is sensible to assume that our understanding of how non-local patterns are codified should depend on our understanding of the motoric genesis of such patterns. Indeed, one can argue that origination should be primary in our understanding of phonologization, because non-local patterns seem unlikely to spontaneously emerge, i.e., come into being without any sensorimotor precursors.

One obstacle in this endeavor is our incomplete knowledge of the extent to which an empirically observed non-local pattern is the product of active mechanisms which operate on long-term memories or is codified directly in lexical memory. To illustrate this distinction, consider the schematic harmony patterns in Table 2. Some non-local patterns, and in particular, many consonant harmonies (see Hansson, 2001), appear to be lexical co-occurrence restrictions in the domain of a lexical root (1) or derivational stem (2). In these cases, it is quite sensible to interpret the pattern as directly encoded in long-term memory: the gestures that are retrieved from memory in association with a word form already conform to the harmonic pattern, and therefore no mechanism is required to generate the harmony in utterance planning. In contrast, other non-local patterns are better understood as actively generated by the production system during the process of planning an utterance. Vowel harmonies and vowel-consonant harmonies may be more likely to be of the active variety than consonant harmonies, because in some cases, these harmonies apply in an inflectional domain (3), i.e., a morphologically complex form that includes inflectional morphology (i.e., tense, aspect, mood, agreement, number, person, etc.). It is worth mention that even productive harmonies involving inflectional morphology might be construed as lexical if we allow for analogical mechanisms to influence the selection of morphs from the lexicon.

**TABLE 2:** Hypothetical non-local patterns which apply in different morphological domains.

**Harmony domain**		
**1. Lexical roots**	**2. Derivational stems**	**3. Inflectional stems**
a. ʃ apa ʃ	a. n ap + an	a. ta p = a s
b. ^∗^ʃapas	b. ^∗^n ap + al	b. ^∗^ta p = æ s
c. sapas	c. l ap + al	c. tæ p = æ s
d. ^∗^sapa ʃ	d. ^∗^l ap + an	d. ^∗^tae p = a s

An important clarification to make here is that there are several senses of locality that may be applied to describe phonological patterns. One sense is based on the conception of speech as a string of symbols – i.e., segments which are arranged in a linear order. Another is based on the idea that the articulatory manifestations of a harmony pattern are continuous in time (Gafos, 1999; Smith, 2018), which is closely related to tier-based analyses in which articulatory features on a tier can spread (Goldsmith, 1979). A third sense is based on the temporal continuity of the motoric mechanisms which give rise to a pattern. We will show in sections “Spreading Arises From Selectional Dissociation” and “Agreement Arises From Leaky Gestural Gating” that the motoric mechanisms which give rise to harmony patterns are always local, even when articulatory manifestations are not. Identifying local mechanisms for the origination of such patterns is desirable because, as some have argued (e.g., Iskarous, 2016) physical laws always specify *local* relationships between variables in space and time, and so there cannot be a truly “non-local” mechanism. To show how these three conceptions of locality apply, Table 3 classifies various assimilatory phonological patterns.

**TABLE 3:** Locality-based classification of origins of assimilatory phonological patterns.

**Pattern**	**Hypothesized mechanisms**	**String locality**	**Articulatory locality**	**Motoric locality**
a. CC assimilation (tautosyllabic)	Gestural blending/overlap	Y	Y	Y
b. VC assimilation (tautosyllabic)		Y	Y	Y
c. CC assimilation (heterosyllabic)	Anticipatory de-gating/delayed suppression	Y	Y	Y
d. VC assimilation (heterosyllabic)		Y	Y	Y
e. V harmony (spreading)			Y	Y
f. VC harmony (spreading)			Y	Y
g. C harmony (spreading)			Y	Y
h. C harmony (agreement)	Subthreshold gestural forces/leaky gating			Y

Our main focus in the following sections is on the last two types of patterns listed in Table 3: spreading harmonies (e–g) and agreement consonant harmony (h). It is nonetheless worthwhile to briefly consider how other types of patterns arise. One of the most cross-linguistically common phonological patterns is assimilation of adjacent sounds which are associated with the same syllable (a, b). Such patterns have been thoroughly examined in the AP/TD framework and can be readily understood through a gestural blending mechanism (Browman and Goldstein, 1990; Gafos, 2002; Gafos and Goldstein, 2012). In the selection-coordination-intention framework, gestures which are associated with the same syllable are co-selected. When co-selected gestures exert forces on the same intentional planning field, the strengths of those forces are blended. When co-selected gestures exert forces on distinct intentional planning fields, overlap of gestural activation can occur without blending coming into play. In either case, the co-activation of gestures can lead to phonologization of new articulatory targets, i.e., changes in the long-term memory specification of gestural-motoric organization associated with a word form.

Assimilatory patterns between sounds associated with different syllables (c, d) must be understood differently from tautosyllabic patterns because the relevant gestures are associated with distinct competitively selected sets of gestures and therefore those gestures are canonically selected in different epochs. We have already shown in section “Selectional Dissociation and Local Coarticulation” how local coarticulation arises from the dissociation of gestural selection from canonical motoric organization. Specifically, internal feedback allows for some gesture or gestures to be promoted early or demoted late. These phenomena result in gestural overlap and constitute an active mechanism for generating assimilatory patterns. Moreover, they can be phonologized as assimilatory phonological alternations in long-term memory. As we argue below, selectional dissociation is also the mechanism via which spreading harmonies emerge.

The main proposal here is that there are two distinct mechanisms via which harmony patterns can arise: selectional dissociation and subthreshold intentional planning. The former gives rise to so-called “spreading” patterns which are not distinct, in a mechanistic sense, from assimilation of adjacent, heterosyllabic sounds. Spreading patterns are articulatorily local, in the sense described above. It is possible that all vowel and vowel-consonant harmonies are of this variety (Hansson, 2001; Nevins, 2010; Van der Hulst, 2011; Smith, 2018), and that some consonant harmonies are as well (Gafos, 1999). The other mechanism – subthreshold intentional planning – is associated with at least *some* consonant harmonies, which are described as “agreement” or “correspondence” patterns (Piggott and Van der Hulst, 1997; Walker, 2000; Hansson, 2001; Rose and Walker, 2011).

The crux of the empirical distinction between spreading vs. agreement amounts to whether there are articulatory manifestations of the relevant gesture during the period of time between the trigger and target segments. Let’s consider a common variety of consonant harmony: coronal place harmony of sibilants. A prototypical example is one in which all sibilants in lexical root have the same anteriority as the last sibilant in the root (see Table 2, example 1). Gafos (1999) argued that a tongue tip constriction location (TTCL) gesture can be active during vocalic or non-coronal consonantal gestures which intervene between the trigger and target, without inducing a substantial auditory perturbation of the sensory consequences of those gestures. In other words, the position of the tongue blade may be physically influenced during the intervening segments, regardless of whether the influence has audible consequences. Indeed, some experimental evidence of this effect was provided in Gafos (1999). In this analysis, there is an articulatory continuity with respect to activation of the relevant TTCL gesture: the pattern is articulatorily local.

However, it has not been demonstrated that all sibilant harmonies exhibit continuous articulatory manifestations of this sort, and in most cases it is impossible to determine if such patterns originated in that manner. Moreover, there are other consonant harmonies which are highly unlikely to have originated from a continuous articulatory manifestation. One example is nasal consonant harmony, in which the nasality of certain classes of consonants must agree in a root or derived stem (see Table 2, example 2). Walker (2000) and Hansson (2001) have pointed out that continuous velum lowering between trigger and target would result all intervening vowels being nasalized and all intervening consonants being nasalized. Yet such nasalization of intervening segments is not observed in nasal consonant harmony (recall that this issue was raised in section “Introduction,” in relation to Figure 3). This argues against conceptualizing nasal consonant harmony as the result of a continuously active gesture: such patterns are articulatorily non-local. The reader should note that nasal consonant harmony is distinct from nasal spreading (Cohn, 1993; Hansson, 2001); in nasal spreading intervening segments *are* nasalized.

Another example of a pattern which is articulatorily non-local is laryngeal feature harmony (Hansson, 2001), where oral stops with different laryngeal features (e.g., aspirated vs. ejective) may not co-occur in some domain. In a gestural framework, aspiration corresponds to a glottal opening gesture and ejection to a combination of glottal closing and laryngeal elevation gestures. It is not physically possible for the glottis to be open or fully closed during intervening vowels or voiced continuant consonants, without substantially influencing the acoustic manifestations of those sounds. Thus laryngeal harmonies are another type of consonant harmony pattern which cannot be readily understood as the result of articulatory continuity/continuous gestural activation.

The impossibility of articulatory continuity in certain harmonies is one motivation for distinguishing between mechanisms for the emergence of spreading and agreement; another is that there are numerous typological differences between patterns analyzed as spreading vs. agreement. In particular, these include differences in (i) blocking and transparency of intervening segments, (ii) morphological domain sensitivity, (iii) prosodic domain sensitivity, (iv) structure preservation, (v) similarity sensitivity, and (vi) directionality biases. Section “Spreading Arises From Selectional Dissociation” shows how spreading/blocking is modeled in the selection-coordination-intention framework, section “Agreement Arises From Leaky Gestural Gating” shows how agreement is modeled, and section “Deriving the Typology of Agreement and Spreading Patterns” addresses the aforementioned typological differences.

### Spreading Arises From Selectional Dissociation

The intention and selection models developed in sections “The Intentional Planning Mechanism” and “Gestural Selection and Intentional Planning” generate spreading via the mechanism of selectional dissociation. Recall from section “Selectional Dissociation and Local Coarticulation” that a gesture which is canonically selected in a given epoch can be anticipatorily selected in an immediately preceding epoch, or the suppression of the gesture can be delayed to occur in a subsequent epoch. In other words, gestural selection can be dissociated from canonical motor set organization, such that gestures may be promoted early or demoted late. In typical circumstances, there are perceptual and contrast-related forces which may prevent anticipatory degating and delayed suppression from occurring too extensively. If the selectional dissociation compromises sensory information which is important for the perceptual recoverability of preceding gestures, it will not be too extensive. Moreover, if an inhibitory gesture [y−] is selected in some epoch, and [y−] is antagonistically related to [x+], then [x+] is unlikely to be anticipatorily promoted or belatedly suppressed in that epoch. However, early promotion or late demotion may not be perceptually or informationally disadvantageous, and may even be advantageous. Thus in the absence of the antagonistic gesture [y−], we would expect that the anticipation or perseveration of [x+] may extend throughout the relevant epoch.

**FIGURE 11:**
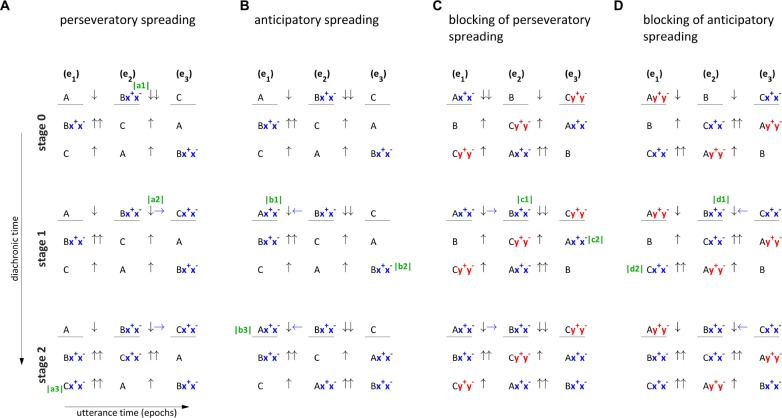
Spreading and blocking selection trajectories. **(A,B)** spreading of [x^+^] and [x^–^] occurs when selection of a gesture is dissociated from the epoch in which it is canonically selected. **(C,D)** Blocking occurs when promotion of an antagonistic gestures [y^–^] and [y^+^] necessitates demotion of [x^+^] and [x^–^] or prevents promotion of these gestures. Horizontal lines in selection trajectories represent the selection threshold. Labels | a1|, | b2|, etc… are referenced in the text.

Selection trajectories for perseveratory and anticipatory spreading are schematized in Figures 11A,B. Labels |a1|, |b2|, etc… are included to facilitate exposition. The examples involve a word form with three competitively selected sets of gestures: A, B, and C. The relevant spreading gestures are a (+)/(−) pair labeled as [x^+^] and [x^–^]. For concreteness, the reader can imagine that A, B, and C are comprised of oral consonantal constriction and vocalic gestures, and that [x^+^] and [x^–^] are excitatory and inhibitory [VEL op] gestures. For the perseveratory spreading pattern in Figure 11A, let’s suppose that on a diachronic timescale there is an initial stage (stage 0) in which the selection trajectory is canonical; specifically, [B], [x^+^], and [x^–^] comprise a set of gestures {Bx^+^x^–^}, which is competitively selected relative to sets {A} and {C}. In the stage 0 trajectory, [x^+^] and [x^–^] are demoted in epoch (e_3_), when [B] is demoted (|a1|). In a subsequent stage (stage 1), the demotion of [x^+^] and [x^–^] is delayed relative to demotion of [B], and hence [x^+^] and [x^–^] remain selected during epoch (e_3_) in which gestures of {C} are also selected (|a2|). This diachronic stage represents an active spreading process, and we conjecture that [x^+^] and [x^–^] can remain in a selected state through each subsequent epoch. The anticipatory version of spreading in Figure 11B is quite similar, except in this case [x^+^] and [x^–^] are promoted early in epoch (e_1_) (see |b1|) and persist in a selected state until gestures in the set they are canonically associated with, {Bx^+^x^–^}, are demoted (|b2|).

It is worth mention that while some spreading patterns have a clear directionality, in others directionality is unclear, or can be analyzed as bidirectional. Moreover, in both anticipatory and perseveratory cases, the spreading can be phonologized in a subsequent diachronic stage, such that [x^+^] and [x^–^] become members of each selection set that is organized upon retrieval of the word form (i.e., |a3| and |b3|). In this case, the selectional dissociation may or may not remain active. If the pattern is observed in productively derived stems or inflectional stems, it is most likely still active. Indeed, it is plausible that spreading can involve iterative phonologization of the relevant feature, such that (i) selectional dissociation perturbs articulation in a temporally adjacent epoch, (ii) the perturbation is phonologized, and then steps (i) and (ii) repeat for another pair of epochs.

An important characteristic of spreading is that it always involves epochs which are contiguous in utterance time. The reason for this is that anticipatory degating and delayed suppression can only extend the period of time in which a gesture is selected; these mechanisms do not involve additional selections or suppressions of a gesture. This restriction is important in accounting for the occurrence of blocking phenomena, which are represented in Figures 11C,D. As explained in section “Selectional Dissociation and Local Coarticulation” selectional dissociations are dependent upon whether there is an antagonistic gesture selected in the epoch which would potentially incorporate a dissociating gesture. This antagonistic gesture is represented as [y−] in Figures 11C,D. For instance, if the trigger gesture [x+] is a [VEL op+] gesture, then the antagonistic gesture [y−] would be [VEL clo−]. Spreading is blocked when it would involve co-selection of [x+] and [y−]. Hence in the anticipatory spreading example of Figure 11C, the gesture [x+] which is selected in (e_1_) can be selected in (e_2_) (label | c1|), but it is demoted in the reorganization to (e_3_) (| c2|) because this reorganization promotes the antagonistic gesture [y−]. In Figure 11D, anticipatory spreading can occur by early promotion of [x+] in (e_2_) (see |d1|), but cannot be promoted in (e_1_) (|d2|) because the antagonistic gesture [y−] is promoted. Thus in Figure 11D [x+] can be selected in (e_2_) but not in (e_1_) when [y−] is selected. Hence spreading and the blocking of spreading are understood as contingent upon whether antagonistic inhibitory gestures are promoted.

In a more detailed sense, the blocking occurs because promotion and demotion are reorganization operations that can enforce mutual exclusivity in the selection of gestures. However, the selectional dissociation mechanism allows for this mutual exclusivity to be violated when the relevant gestures are not strongly antagonistic. For current purposes it is sufficient to interpret the sensitivity of reorganization to antagonistic relations as categorical restriction on reorganizations: if [y^–^] is promoted, [x^+^] must be demoted and cannot be promoted. Thus it is only when no [y^–^] gesture is selected that [x^+^] can be selected in a dissociated manner.

### Agreement Arises From Leaky Gestural Gating

Whereas spreading is understood to arise from selectional dissociations, agreement patterns are modeled here as a consequence of sub-selection level gestural forces on intentional fields. Recall from section “Sub-selection Intentional Planning and Anticipatory Posturing” that when the gestural force gating function is leaky, a gesture which is not selected can exert a substantial force on an intentional field. This leaky gating mechanism was previously used to account for anticipatory posturing prior to production of a word form. There is no obvious reason why such a mechanism should not operate during epochs of production as well, and if that occurs, its effects can generate an agreement pattern. Moreover, this active agreement pattern has the potential to become phonologized via the Ohalan hypocorrective mechanism.

**FIGURE 12:**
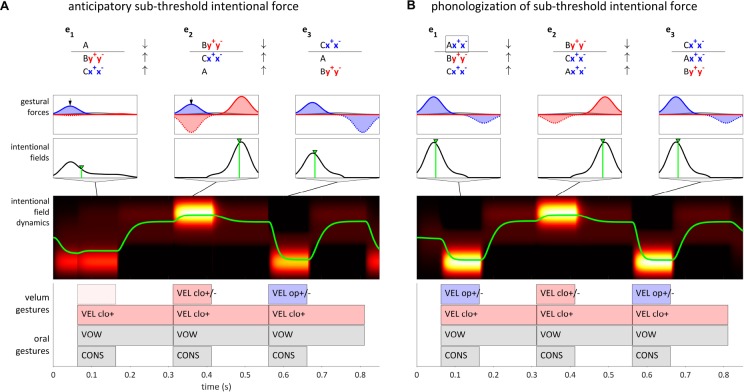
Leaky gating as a mechanism for the emergence of agreement patterns. Intentional field dynamics are shown as a distribution of field activation which evolves over time, where brighter shade represents more activation, and the green line represents the current centroid of activation. **(A)** [x+], a [VEL op+] gesture with leaky gating, exerts a force on an intentional field prior to its selection. An overt influence on articulation is not observed when an antagonistic gesture is selected. **(B)** The influence of [x+] on the intentional field is phonologized as selection of [x+] and [x–] in an earlier epoch.

An example of an active agreement pattern is shown in Figure 12A for a word form comprised of three sets of gestures, {A}, {By^+^y^–^}, and {Cx^+^x^–^}. The gestures [x^+^] and [y^–^] are antagonistic. With leaky gestural gating, [x^+^], which is selected in (e_3_), exerts substantial forces on an intentional field in epochs (e_1_) and (e_2_). However, during epoch (e_2_) in which the antagonistic gesture [y^–^] is selected, the force that [x^+^] exerts on the intentional field is canceled by the inhibitory force from the antagonistic gesture [y^–^]. During epoch (e_1_), no gesture which is antagonistic to [x^+^] is selected, and thus the influence of [x^+^] on the intentional field will be manifested articulatorily. In such a situation, we see that the gestures selected in (e_2_) are transparent to the harmony pattern. A concrete instantiation of this example would be a phonological form /ba.sa.na/ which exhibits nasalization of the initial consonant, [masana]. We imagine that [x^+^] is a [VEL op+] gesture associated with the /n/, and that [y^–^] is an antagonistic [VEL clo−] gesture associated with /s/ and the vowel /a/. The phonetic precursors of the non-local agreement pattern can arise if [y−] is not selected in association with /p/.

On a diachronic timescale, we can imagine that the sub-selection influence of [x^+^] can be phonologized, in that the composition of the set of gestures selected in (e_1_) is reinterpreted by speakers as including the gesture [x^+^] along with [x^–^]. This circumstance is shown in Figure 12B. At this point, the sub-selection influence of [x+] may or may not remain present. Ultimately, what this model holds is that the articulatory precursors of an agreement pattern can arise between segments whenever there is no antagonist (of the triggering gesture) that exerts forces on the relevant intentional field, and as long gating of the triggering gesture is leaky. Hence antagonistic gestures block spreading harmonies, but cause transparency in agreement harmonies.

### Deriving the Typology of Agreement and Spreading Patterns

If the proposed distinction between mechanisms of spreading and harmony is useful, it should help us make sense of various typological differences between consonant harmony and vowel harmony, which a number of researchers have argued are associated with agreement and spreading, respectively (see Hansson, 2001; Rose and Walker, 2011). Table 4 lists some of the differences between agreement and spreading. It is worth emphasizing that if there is only one mechanism whereby long-distance phonological patterns arise, then these differences are almost entirely inexplicable, and must therefore be seen as accidental. Thus a model which can account for them is highly desirable.

**TABLE 4:** Typological differences between agreement and spreading patterns.

	**Agreement**	**Spreading**
Blocking	Never blockable	Blockable
Transparency	Intervening segments always transparent	Intervening segments usually not transparent
Morphological domain sensitivity	Restricted to root or derivational domain	Can occur in inflectional domain
Prosodic domain sensitivity	Never	Common
Structure preserving	Always	Not necessarily
Similarity sensitivity	Always	Not sensitive to similarity
Directionality	Anticipatory or stem-controlled	Anticipatory, perseveratory, or stem-controlled

One of the most telling differences between agreement and spreading is that agreement is never blocked by intervening segments, while spreading is blockable (Hansson, 2001; Rose and Walker, 2011). This difference falls out straightforwardly from the models in sections “Spreading Arises From Selectional Dissociation” and “Agreement Arises From Leaky Gestural Gating.” Blocking occurs when promotion of an inhibitory gesture (y^–^) necessitates the demotion of an antagonistically related excitatory gesture (x^+^). Examples of blocking in spreading patterns were provided in section “Spreading Arises From Selectional Dissociation,” Figures 11C,D. Blocking is observed in spreading harmonies because spreading harmonies arise from anticipatory promotion or delayed demotion of a source gesture; in other words, spreading is blockable because spreading derives from gestural selection, which is constrained by antagonistic relations between gestures. In contrast, blocking is never observed in agreement patterns because agreement patterns do not arise from gestural selection. Instead, agreement arises from leaky gating of a gesture with sub-selection level excitation; blocking does not occur in agreement patterns because the relevant gestural system need not be selected in order to influence the state of the vocal tract.

Along these same lines, intervening segments which are not targets of an agreement pattern are always “transparent” in agreement patterns, in the sense that they involve the selection of an antagonist whose influence on the relevant intentional field outweighs the influence of the triggering gesture. Intervening segments in a spreading pattern must either block the selection of the dissociated gesture or allow selection of that gesture, in which case those segments will exhibit physically observable characteristics of the relevant articulatory state. Thus the differences in blockability and transparency of agreement and spreading patterns fall out naturally from the hypothesized difference in mechanisms. For example, in nasal spreading harmony, intervening vowels which become nasalized typically lack contrastive nasalized vowel counterparts. Hence we can infer that in such cases there is no [VEL clo−] antagonist selected with the vowels which would prevent the early promotion or late demotion of [VEL op+].

Agreement is almost always morphologically restricted to a root or derivational morphological domain, whereas spreading often extends to inflectional morphs and even clitics (Hansson, 2001: 430). As Hansson (2001: 430) puts it, “consonant harmony is never postlexical.” Because we have not developed an explicit model of the role of morphological domains in gestural-motoric organization, a detailed analysis of this typological distinction cannot be presented. Nonetheless, to explain why agreement never seems to involve inflectional domains, we might conjecture that the reorganization operations associated with inflectional forms always enforce strong gestural gating: during epochs in which an inflectional form is selected, all gating functions are non-leaky. This would account for why agreement never extends to inflectional morphs.

Agreement is never sensitive to stress or other metrical structure, and is never bounded by prosodic domains such as the foot; in contrast, such prosodic domain restrictions are common for spreading patterns, such as vowel harmonies and vowel-consonant harmonies (Hansson, 2001; Rose and Walker, 2011). This difference can be interpreted with the idea that domains such as the prosodic word are associated with the selection of accentual gestures (Tilsen, 2018a, 2019), in conjunction with the idea that selection of accentual gestures can influence the promotion and demotion of articulatory gestures. Accentual gestures specify F0 and/or intensity targets, and are associated with stress (i.e., metrical structure) as well as intonation (pitch accents). If we assume that the selection of an accentual gesture can enhance the likelihood that speakers select a gesture which is antagonistic to a spreading gesture, or at least augment the antagonism, then we can generate patterns in which spreading harmonies are restricted to a particular prosodic domain. In contrast, this hypothesized effect of selecting an accentual gesture will have no bearing on the mechanism whereby agreement patterns arise, because such patterns are not contingent on selection of the triggering gesture.

Another typological difference is that agreement is always structure-preserving, in that agreement patterns never give rise to new classes of segments (Hansson, 2001). In contrast, spreading can and often does result in an expansion of the segmental inventory. To account for this, we must interpret the difference as a consequence of the phonologization of agreement and spreading patterns. When the sub-threshold gestural influence on an intentional field is reinterpreted as selection of the triggering gesture, that reinterpretation is constrained to result in selection of a set of gestures which already exists in the inventory of such sets in a given language. What makes spreading different is that the triggering gesture which is phonologized as a member of another selection set is *already selected* during the epoch governed by that set. Thus any prohibitions on reinterpretations which result in new sets of gestures in the inventory are weaker.

Agreement harmonies always involve segments which are similar, while spreading patterns do not necessarily involve similar segments. For example, nasal consonant harmonies are always restricted to a subclass of consonants – e.g., coronal sonorants – such that consonants not in this class are transparent to the harmony. This is expected if featurally similar segments are more likely to lack an antagonistic gesture which would oppose the subthreshold influence of the triggering gesture. It is worth noting that similarity appears to be factor in speech errors as well: segments which share more features are more likely to participate in substitutions and exchanges than segments with fewer features in common (Fromkin, 1971; Nooteboom, 1973; Shattuck-Hufnagel, 1979; Frisch, 1997). In contrast, the relations between triggers and targets in spreading harmonies are not expected to be constrained by featural similarity because antagonistic gestures block spreading; in the absence of this blocking any segment from an adjacent epoch can be influenced by dissociated selected.

Finally, agreement harmonies are predominantly anticipatory, and those cases which are not anticipatory can be analyzed as instances of stem-control (Hansson, 2001: 467). In contrast, spreading harmonies show a weaker bias for anticipatory directionality. This anticipatory bias in agreement patterns suggests that the subthreshold influence of a gesture may be stronger before the gesture is selected than after the gesture has been suppressed. This makes sense if we assume that suppression causes the excitation of the gesture to be lower than it was prior to selection. The force exerted on an intentional field is always a function of gestural excitation, and presumably even leaky parameterization of the gating function does not allow gestures with very low excitation to have strong influences on intentional fields. Our analysis of spreading, in contrast, does not hinge on the sub-threshold excitation of gestures, and therefore no similar bias is expected.

## General Discussion and Conclusion

In this paper we presented a new model of how the target state of the vocal tract is controlled in the planning and production of speech. Specifically, we argued that for each parameter of vocal tract geometry in the Articulatory Phonology/Task Dynamics model, there is a one-dimensional field – an *intentional planning field* – in which a distribution of activation determines the current target value of that parameter. These intentional planning fields receive distributions of both excitatory and inhibitory input from gestural systems, and on that basis we distinguished between excitatory gestures and inhibitory gestures. In this expanded conception, we distinguished between *dynamic targets*, which vary continuously and are derived from integrating the distribution of activation in an intentional field, and *gestural targets*, which are associated with distributions of excitatory or inhibitory forces that gestures exert on the activation of intentional fields. Furthermore, the proposed model of intentional planning was integrated with the selection-coordination framework (Tilsen, 2016, 2018b), in which sequencing of syllable-sized sets of gestures is accomplished via a competitive selection mechanism. The competitive selection mechanism is conceptualized as the organization of gesture sets in a step potential, in which selection sets are iteratively promoted and demoted.

There are several ways in which the model presented here complicates our understanding of speech, and thus it is important to establish why such complications are warranted. In general, when two models fare equally well in describing the same empirical phenomena, we should prefer the simpler model. But if the more complicated model accounts for a wider range of empirical phenomena than the simpler one, we must weigh the advantages of broader empirical coverage against the disadvantage of greater model complexity. In the current case the expanded empirical coverage outweighs the increase in complexity and therefore justifies the model. There are also ways in which the proposed model is simpler than the standard AP/TD model, and these constitute arguments in its favor. To elaborate on these points, we review the phenomena that the selection-coordination-intention model addresses.

First, we observed in section “Introduction” that there are aspects of control over the state of the vocal tract that gestural scores do not explicitly represent. Specifically, we showed that there are two alternative ways of conceptualizing how a consonantal constriction is released. On one hand, the standard AP/TD model accomplishes releases via the influence of a neutral attractor on model articulators. Crucially, we noted that in order to avoid unwanted influence of the neutral attractor during periods of time in which gestures are active, the AP/TD model competitively gates the influence of the neutral attractor on model articulators. The competitive gating amounts to turning the neutral attractor on and off in a way that is precisely locked to the activation of gestures and contingent on the model articulators that are influenced by those gestures. Alternatively, we suggested that releases of constrictions can be driven by active gestures. Despite increasing the number of gestures that are involved in production of a word form, this alternative is simpler in that it does away with the need to competitively gate the neutral attractor in a way that is precisely timed to gestural activation. A nice consequence of this view is that we do not need to posit *ad hoc* constructs such as a default modal-voicing state of the glottis during speech: all movement is driven by intentional planning fields which evolve continuously in time. The competitive gating account is also somewhat unsatisfactory from a conceptual standpoint, in that it requires a mechanism which is sensitive not only to the tract variables which gestures are associated with, but also the model articulators that are used to effect changes in those tract variables. In other words, the competitive gating of the neutral attractor, because it applies to model articulators instead of tract variables, constitutes an additional layer of mechanistic complexity in the AP/TD model. The proposed alternative is simpler because the neutral attractor is reinterpreted as a set of constant, relatively weak forces on intentional planning fields; no dynamic modulation or gating of this force is necessary.

Second, in sections “Empirical Evidence for Intentional Planning” and “The Inadequacy of Gestural Blending” we considered the empirical phenomena of assimilation and dissimilation between contemporaneously planned targets. It was argued that the standard AP/TD model cannot generate either sort of pattern, because in that model gestures only have influences on the vocal tract when they are active. In distractor-target paradigms where assimilatory and dissimilatory patterns are observed, the distractor is never produced, hence the corresponding gesture should not be active and should have no influence on production. Furthermore, in the standard model, dissimilatory patterns would require a problematic form of gestural gating in which blending negatively weights the influence of the distractor. In contrast, the intentional planning model readily accounts for both assimilatory and dissimilatory patterns, without requiring gestural activation or unusual gating. This is accomplished by hypothesizing that gestures which are not selected can exert forces on intentional planning fields, and that those forces can be excitatory and/or inhibitory. Although this account is more complex, it succeeds in generating the empirical patterns.

Third, in the section “Sub-selection Intentional Planning and Anticipatory Posturing” we considered the phenomenon of anticipatory posturing, which involves the partial assimilation of vocal tract posture to targets of an upcoming response. The standard AP/TD model cannot account for this phenomenon without fairly *ad hoc* stipulations, such as positing multiple targets for gestures, new gestures, or special dynamics of gestural gating. The selection-coordination-intention model generates anticipatory posturing through influences of non-active (i.e., excited but not selected) gestures on intentional planning fields. These subthreshold influences are governed by parameterization of the gestural gating function, which determines the strengths of the forces exerted by excited gestures on intentional fields. It was shown that leaky gating allows such influences to be non-negligible, and that blending those influences with the constant influence of the neutral attractor accounts for the partially assimilatory quality of anticipatory posturing.

Fourth, in section “The Origins of Non-local Phonological Patterns,” we examined two varieties of non-local phonological patterns, spreading harmony and agreement harmony. It was shown that these two varieties of harmony can be understood to originate through distinct mechanisms. Spreading harmonies were understood to arise from selectional dissociations in which anticipatory degating (i.e., early promotion) or delayed suppression (i.e., late demotion) cause a gesture to be selected in an epoch other than the one in which it is canonically selected. One prediction of this account that could be readily tested is that (non-phonologized) spreading will be less extensive when external feedback plays a greater role in gestural selection and suppression, i.e., in slower, more careful speech. In contrast, agreement harmonies were understood to arise from leaky gating of gestural forces on intentional fields. The role of leaky gating in both anticipatory posturing and the origination of agreement patterns predicts that there may be correlation between the extent to which a speaker may exhibit an anticipatory articulatory posture in some tract variable and their ability to learn an agreement harmony involving that that tract variable.

Importantly, the proposed mechanisms account for a key phenomenological difference between spreading and agreement: the possibility of blocking. Spreading harmonies can be blocked because they hinge on selection of a gesture, and the selection of a given gesture is prohibited when an antagonistic gesture is selected. Agreement harmonies are never blocked because they do not require selection of the relevant gesture; intervening segments are thus always transparent. Furthermore, we discussed how a number of typological differences between spreading and agreement could be understood in the context of the model. These involved the sensitivity of such patterns to morphological and prosodic domains, structure preservation, similarity sensitivity, and directionality biases. The standard AP/TD model does not provide two distinct mechanisms for the origins of spreading and agreement, and so there is no straightforward way to understand the typological differences between such patterns.

In sum, the selection-coordination-intention model, while more complicated than standard AP/TD, addresses a broader range of empirical phenomena: assimilation/dissimilation of contemporaneously planned targets, anticipatory posturing, and spreading/agreement harmonies. A desirable consequence of the model is that agreement harmonies can be viewed as the result of a motoric mechanism which operates locally, i.e., involves continuous influence on an intentional field. This makes it unnecessary to stipulate non-local mechanisms in the utterance-timescale genesis of phonological patterns. The model also simplifies our understanding of control over the vocal tract by eliminating the need for a special blending mechanism involving the neutral attractor. The primary downside of the selection-coordination-intention model is the need for more detailed specification of the gestures that are involved in production of a word form, including a dissociation between excitatory and inhibitory gestures. An outstanding issue is whether there are undiscovered generalizations about when both excitatory and inhibitory gestures need to be specified, and when it is possible to specify only one of these. Future work should explore this question.

## Author Contributions

The author confirms being the sole contributor of this work and has approved it for publication.

## Conflict of Interest

The author declares that the research was conducted in the absence of any commercial or financial relationships that could be construed as a potential conflict of interest.

## Footnotes

1 Here an explicit model of conceptual-syntactic organization is not provided, but see Tilsen (2019) for a model which in many ways parallels the model of gestural-motoric organization developed here. Although in this paper we associate a pattern of gestural-motoric organization with “word forms,” it is more accurate to associate such patterns of organization with prosodic words, which can include phonologically bound forms such as clitics.

2 It is assumed that experiences from producing and perceiving words contribute to changes in microscopic state variables of the nervous system (e.g., synaptic efficacy and connectivity), which determine macroscopic properties of the production system (i.e., g–m resonances and organization). These macroscopic properties are “lexical knowledge” in that sense that they are associated with semantic concepts and derive from systems which change relatively slowly over time. It is beyond the scope of this paper to develop more detailed microscopic and macroscopic models of these long-term memories.
